# Acquired *MET* Exon 14 Alteration Drives Secondary Resistance to Epidermal Growth Factor Receptor Tyrosine Kinase Inhibitor in *EGFR*-Mutated Lung Cancer

**DOI:** 10.1200/PO.19.00011

**Published:** 2019-05-10

**Authors:** Ken Suzawa, Michael Offin, Adam J. Schoenfeld, Andrew J. Plodkowski, Igor Odintsov, Daniel Lu, William W. Lockwood, Maria E. Arcila, Charles M. Rudin, Alexander Drilon, Helena A. Yu, Gregory J. Riely, Romel Somwar, Marc Ladanyi

**Affiliations:** ^1^ Memorial Sloan Kettering Cancer Center, New York, NY; ^2^ University of British Columbia, Vancouver, British Columbia, Canada

## INTRODUCTION

Novel resistance mechanisms to epidermal growth factor receptor (EGFR) tyrosine kinase inhibitors (TKIs) in *EGFR*-mutant lung cancer continue to be defined.^1^ With the recent approval of the third-generation EGFR TKI osimertinib as first-line treatment, and expanded use of large-panel next-generation sequencing (NGS)–based testing, many novel resistance mechanisms are emerging. Resistance to osimertinib can result from on-target mechanisms, such as the acquisition of second-site *EGFR* mutations, or from the activation of off-target mechanisms or bypass pathways, including acquired oncogenic fusions of *RET*, *ALK*, *BRAF*, and *FGFR*^1,2^ and amplification or mutation of *HER2*, *BRAF*, *MEK*, *KRAS*, *PIK3CA*, and *MET*.^3^

*MET* amplification is reported in 5% to 22% of EGFR TKI resistance.^1,4^ Preclinical studies have shown that acquired resistance to EGFR TKIs resulting from *MET* amplification can be reversed by combined therapy with EGFR and MET inhibitors.^4^ *MET* exon 14 skipping alteration (*MET*ex14) is present in 3% to 4% of lung adenocarcinomas,^5^ and the MET receptor lacking exon 14 shows decreased protein turnover because of loss of the ubiquitination site encoded by exon 14, resulting in aberrant MET activation and oncogenesis.^6^ In preclinical and clinical studies, responses to MET inhibitors, such as crizotinib, have been reported in patients with lung cancer with *MET*ex14 as a primary driver.^6-11^ However, it has not been previously implicated in acquired resistance to EGFR-TKIs. In this study, we used targeted NGS with Memorial Sloan Kettering Integrated Mutation Profiling of Actionable Cancer Targets (MSK-IMPACT),^12^ immunohistochemistry, cell-free DNA testing, and fluorescence in situ hybridization to evaluate acquired resistance mediated by *MET*ex14. Furthermore, we used in vitro functional studies to establish *MET*ex14 as a novel mechanism of acquired resistance to EGFR TKIs.

We used MSK-IMPACT, a large-panel NGS assay, to detect mutations, copy-number alterations, and select gene fusions involving up to 468 cancer-associated genes.^12^ *MET*ex14 was introduced into PC9 and H1975 cells as follows. Briefly, full-length *MET*ex14 was polymerase chain reaction amplified and subcloned into pLenti-CMV-blast lentiviral vector (plasmid 17451; Addgene, Cambridge, MA). The lentiviral plasmids were cotransfected with packaging plasmids into HEK 293 T cells using FuGENE HD (Promega, Madison, WI), and lentiviruses were generated. Cells were infected with lentivirus-expressing *MET*ex14 cDNA, followed by selection with blasticidin (20 µg/mL) for 8 days. The Data Supplement provides more detailed methods.

## CASE REPORT

**FIG 1.**
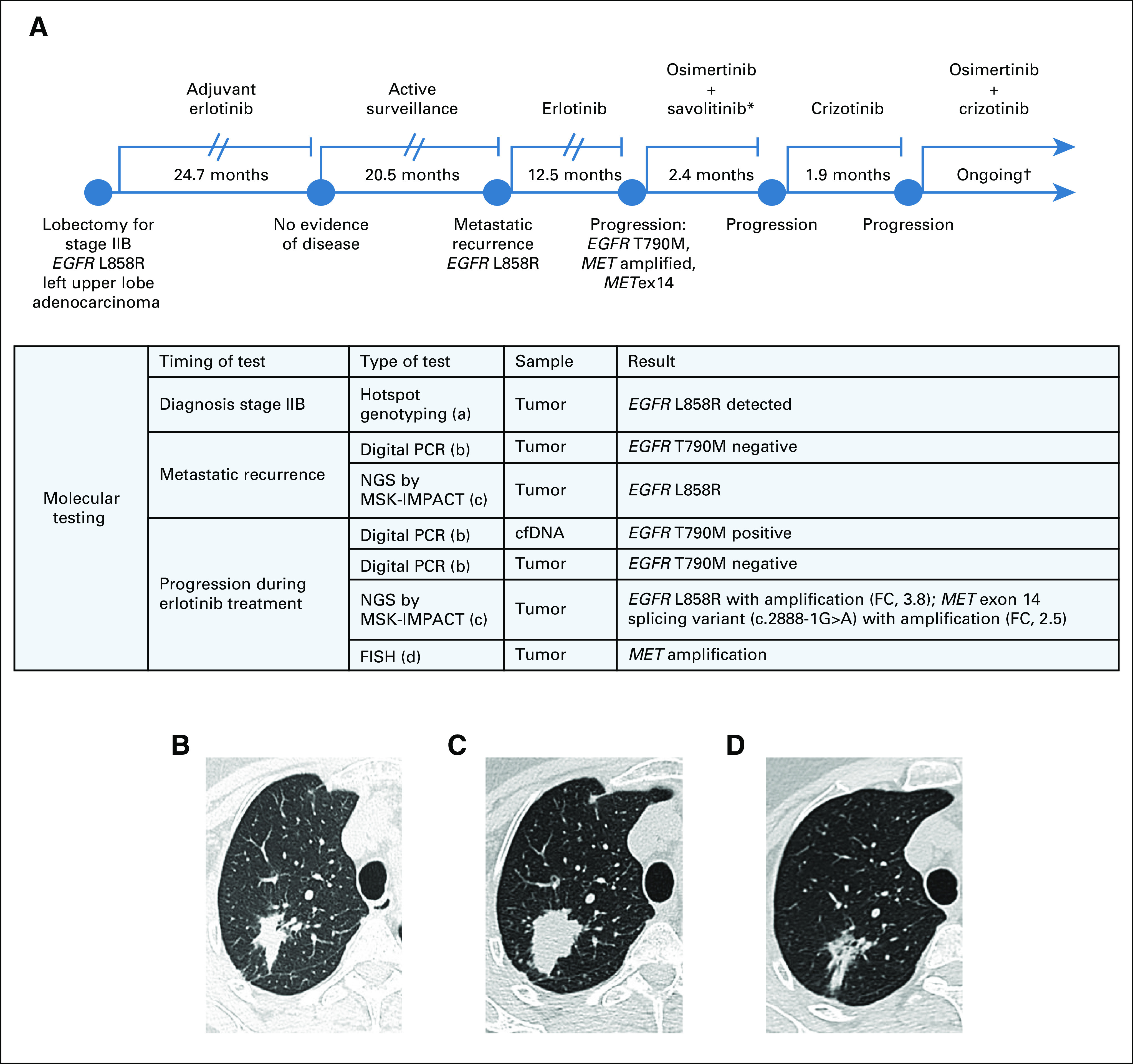
Case summary. (A) Summary of disease course, therapy, and molecular findings. (a) Sequenom mass spectrometry genotyping (Data Supplement). (b) Digital polymerase chain reaction (PCR) for *EGFR* T790M on tissue and/or cell-free DNA (cfDNA). (c) Memorial Sloan Kettering Integrated Mutation Profiling of Actionable Cancer Targets (MSK-IMPACT) large-panel next-generation sequencing (NGS) assay. (d) Fluorescence in situ hybridization (FISH) analysis. (B-D) Representative images showing (B) baseline scan (at time of progression during osimertinib monotherapy), (C) response to crizotinib monotherapy, and (D) response to combined crizotinib and osimertinib therapy. The patient continued to show stable disease 10 months after initiation of combination therapy. FC, fold change. (*) The patient initially received 1.4 months of combination osimertinib and savolitinib in a clinical trial, but treatment was changed to monotherapy with osimertinib because of intolerable toxicity. (†) As of 10 months of ongoing treatment with osimertinib and crizotinib.

A 73-year-old woman who had never smoked presented with lung adenocarcinoma, which was diagnosed via bronchoscopy with biopsy of the left upper lobe, and underwent a left upper lobe lobectomy and lymph node dissection, which showed a stage IIB (pT2bN0M0) poorly differentiated adenocarcinoma. Sequenom mass spectrometry^13^ revealed an *EGFR* L858R mutation, and the patient was administered adjuvant erlotinib (100 mg daily).^14^ After 24.7 months of erlotinib, given no recurrence, adjuvant therapy was discontinued (Fig 1A). The patient was observed for 20.5 months, when imaging revealed new bilateral pulmonary nodules, right-sided paratracheal lymphadenopathy, and a sclerotic T11 lesion. Right upper lobe biopsy confirmed recurrent disease, and MSK-IMPACT testing showed the presence of *EGFR* L858R without *EGFR* T790M mutation. The patient restarted erlotinib (100 mg daily) with clinical and radiologic response for 12.5 months, at which time computed tomography revealed an increase in the dominant right upper lobe mass. Fluorescence in situ hybridization of right upper lobe biopsy material revealed *MET* amplification, and cell-free DNA testing^15^ was positive for *EGFR* T790M. MSK-IMPACT revealed an *EGFR* L858R mutation, no evidence of *EGFR* T790M, and a new *MET*ex14 (c.2899G>A) alteration and *MET* amplification (fold change, 2.5; Fig 1A; Appendix Table A1). Therapy was changed to osimertinib with savolitinib daily (ClinicalTrials.gov identifier: NCT02143466) for 1.4 months, after which savolitinib was stopped because of toxicity and single-agent osimertinib 80 mg daily was continued. Progressive disease in the lung was noted after 2.4 months of osimertinib (Fig 1B). Crizotinib 250 mg twice daily was then administered for 1.9 months, at which time further pulmonary progression of disease was noted (Fig 1C). Treatment was changed to combination osimertinib (80 mg daily) with crizotinib (250 mg twice daily). The combination was tolerated without any report of toxicity. At follow-up 2.3, 4.6, and 7.7 months after starting combination therapy, she had ongoing clinical benefit and stable disease by RECIST (version 1.1; −12.2% response; Fig 1D). The patient continued to receive combination therapy with durable clinical and radiographic benefit for more than 9 months.

**FIG 2.**
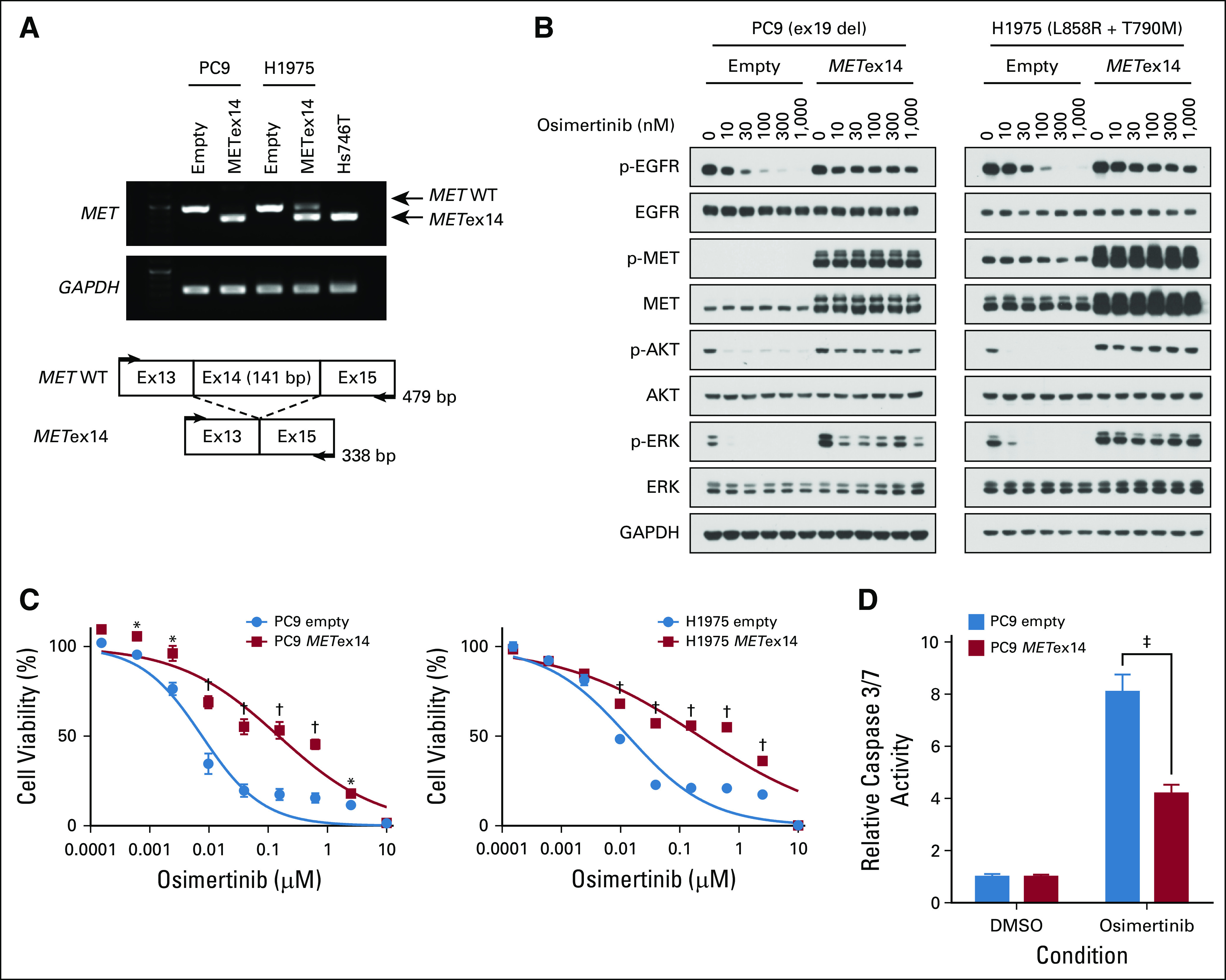
*MET* exon (ex) 14 mutations mediate resistance to epidermal growth factor receptor (EGFR) tyrosine kinase inhibitors in *EGFR*-mutant non–small-cell lung cancer cells. (A) Reverse transcriptase polymerase chain reaction confirmed expression of *MET* exon 14 skipping alteration (*MET*ex14) in the PC9 *MET*ex14 and H1975 *MET*ex14 cell lines. cDNA from Hs746T (gastric cancer cell line with *MET*ex14) was used as a control for *MET*ex14. (B) Cells were treated with increasing concentrations of osimertinib for 3 hours, and lysates were subjected to immunoblotting. (C) Cells were treated with osimertinib for 96 hours, and then growth was determined. Each condition was assayed in eight-replicate determinations, and data represent the mean ± SE of three independent experiments. (D) Caspase 3/7 enzymatic activity was analyzed in cells that were treated with osimertinib (1 µM) for 48 hours. Each condition was assayed in triplicate determinations; data were normalized for cell number by measuring cell viability and shown relative to the control group (mean ± standard deviation). DMSO, dimethyl sulfoxide; GAPDH, glyceraldehyde 3-phosphate dehydrogenase; p, phosphorylated; WT, wild type. (*) *P* < .05. (†) *P* < .01. (‡) *P* < .001.

To define the role of *MET*ex14 in mediating resistance to EGFR TKIs, we generated two isogenic *EGFR*-mutant non–small-cell lung cancer (NSCLC) cell models using PC9 (exon 19 deletion) and H1975 cells (L858R and T790M) by transduction with lentiviral vectors driving expression of *MET*ex14 (Fig 2A). Western blot analysis showed that phosphorylation of EGFR and its downstream effectors AKT and ERK was inhibited by osimertinib in PC9 cells transduced with empty plasmids (PC9 empty), but phosphorylation of EGFR, *MET*ex14, and downstream effectors remained unaffected by osimertinib treatment in PC9 *MET*ex14 cells (Fig 2B). Notably, *MET*ex14 expression correlated with upregulation of phosphorylated EGFR in PC9 *MET*ex14 cells. In cell viability assays, the presence of *MET*ex14 reduced sensitivity to osimertinib by approximately 20-fold (half maximal inhibitory concentration: PC9 empty, 7.7 nM; PC9 *MET*ex14, 150.8 nM; Fig 2C). Similar results were observed with H1975 models in western blotting and cell viability assay (half maximal inhibitory concentration: H1975 empty, 13.4 nM; H1975 *MET*ex14, 216.7 nM; (Figs 2B and 2C). PC9 *MET*ex14 cells showed a reduction in osimertinib-induced caspase 3/7 activation compared with PC9-empty cells (*P* < .001; Fig 2D). Together, these results indicate that *MET*ex14 induces resistance to osimertinib in *EGFR*-mutant NSCLC cells.

**FIG 3.**
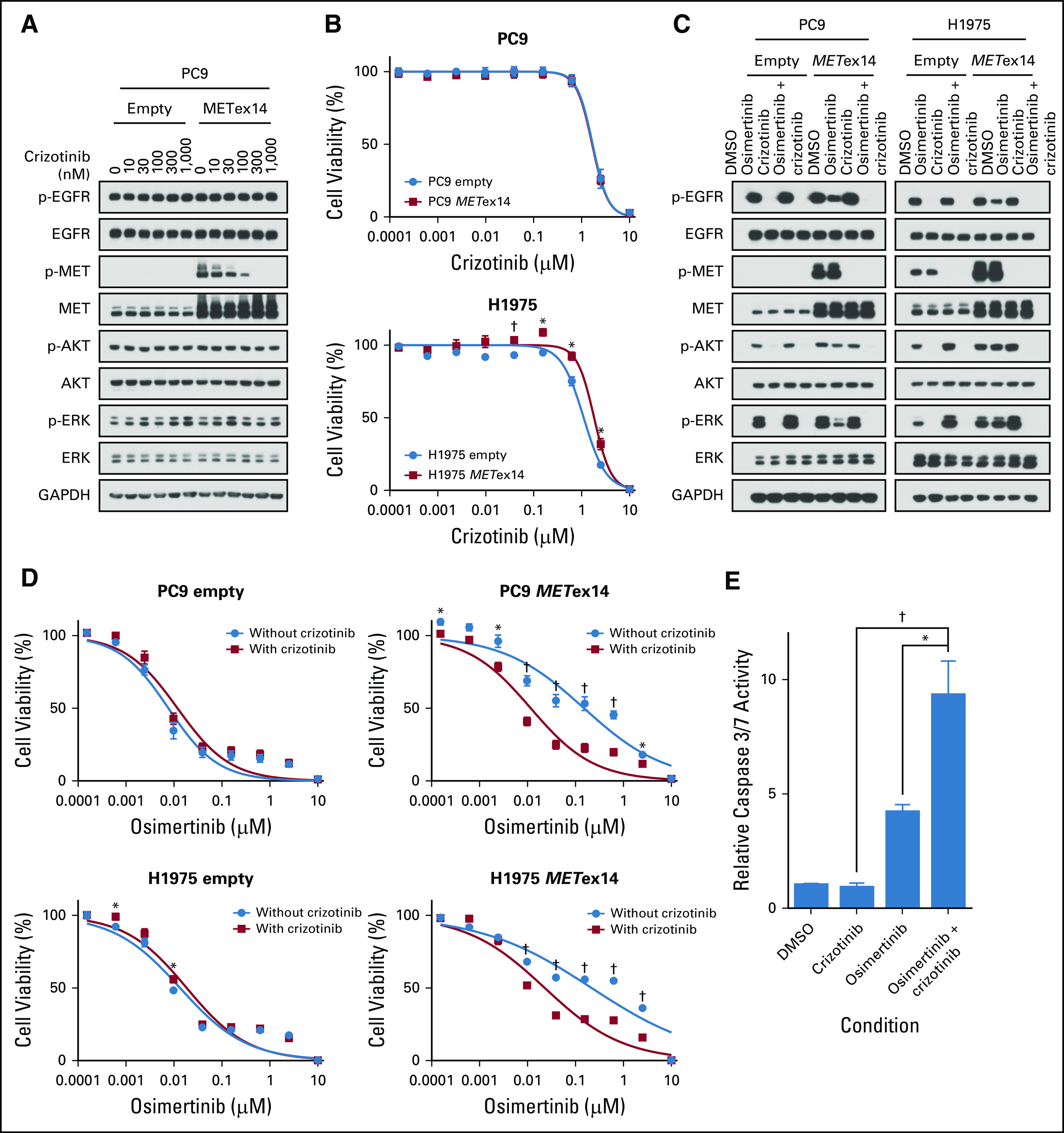
Combination treatment with epidermal growth factor receptor (EGFR) and MET inhibitors is effective against *MET* exon 14 skipping alteration (*MET*ex14) –induced drug resistance in *EGFR*-mutant non–small-cell lung cancer cells. (A) Cells were treated with increasing concentrations of crizotinib for 3 hours, and lysates were subjected to immunoblotting. (B) Cells were treated with osimertinib for 96 hours, and then growth was determined. Each condition was assayed in eight-replicate determinations, and data represent the mean ± SE of three independent experiments. (C) Cells were treated with osimertinib (1 µM), crizotinib (1 µM), or a combination of the two agents for 3 hours, and lysates were subjected to immunoblotting. (D) Cells were treated with osimertinib in the presence or absence of 200 nM of crizotinib for 96 hours. Each condition was assayed in eight-replicate determinations, and data are representative of three independent experiments (mean ± SE). (E) Caspase 3/7 activity was analyzed in PC9 *MET*ex14 cells that were treated with osimertinib (1 µM), crizotinib (200 nM), or a combination of osimertinib (1 µM) and crizotinib (200 nM) for 48 hours. Each condition was assayed in triplicate determinations, and data were normalized for cell number by measuring cell viability and are shown relative to the control group (mean ± standard deviation). DMSO, dimethyl sulfoxide; GAPDH, glyceraldehyde 3-phosphate dehydrogenase; p, phosphorylated. (*) *P* < .05. (†) *P* < .01.

We next investigated whether *MET*ex14-mediated resistance to EGFR TKIs could be overcome by combination therapy with EGFR and MET inhibitors. As expected, crizotinib inhibited *MET*ex14 phosphorylation in PC9 *MET*ex14 cells; however, phosphorylation of EGFR, AKT, and ERK remained largely unchanged (Fig 3A), suggesting that EGFR is still signaling effectively in PC9 *MET*ex14 cells. Similarly, crizotinib was ineffective at modulating growth of *EGFR*-mutated cell lines, with or without *MET*ex14 expression (Fig 3B). However, a combination of osimertinib and crizotinib inhibited activation of EGFR, MET, AKT, and ERK (Fig 3C). Moreover, addition of crizotinib restored the growth inhibitory effects of osimertinib in PC9 *MET*ex14 cells (Fig 3C). Identical results were observed in the H1975 model (Figs 3C and 3D). In agreement with these results, dual inhibition of EGFR and MET caused significantly higher activation of caspase 3/7 (Fig 3E).

## DISCUSSION

Our study highlights the importance of serial and diverse molecular analyses, including NGS, to evaluate acquired alterations in the post-TKI setting. Here, we show how acquired *MET*ex14 mediated resistance to osimertinib. Although the patient did not respond to MET-targeted therapy alone, the patient continued to have a durable clinical response to combination osimertinib and crizotinib, with stable disease by RECIST criteria and without notable toxicity. Our functional data were consistent with these clinical observations. We found that expression of *MET*ex14 in NSCLC cell lines with activating *EGFR* mutation resulted in resistance to osimertinib. Crizotinib restored sensitivity to EGFR TKIs; however, crizotinib alone was not enough to suppress growth.

Two previous reports have demonstrated co-occurrence of *EGFR* and *MET*ex14 mutations. In the first report, three (0.2%) of 1,590 patients with NSCLC harbored concomitant *EGFR* and *MET*ex14 mutations, one of whom received combination treatment with MET- (volitinib) and EGFR-targeted therapies (gefitinib), yielding a partial response.^16^ The second report noted one patient case of sarcomatoid carcinoma with *EGFR* and *MET*ex14 alterations.^17^ In our MSK-IMPACT testing experience of 866 patient cases of *EGFR*-mutant lung adenocarcinomas (as of October 15, 2018), only two patients showed this combination of alterations: the patient described here, with acquired resistance, and a patient in whom the alterations were present in separate primaries at diagnosis.

The clinical benefit of the combination of MET- and EGFR-targeted therapies in patients with NSCLC with acquired *MET* amplification–mediated resistance to EGFR TKIs has been explored in clinical trials, with varying tolerability dependent upon the agents being combined.^18^ Our findings provide a rationale for future clinical evaluation of this combination approach, given its tolerability and efficacy in this case, for patients with *EGFR* and *MET*ex14 mutations. Furthermore, given the recent reports of secondary-site mutations in the *MET* kinase domain, such as D1228N/V and Y1230C, as mechanisms of acquired resistance to crizotinib in patients with *MET*ex14,^19^ it is plausible that these secondary *MET* mutations will also emerge as mechanisms of resistance to the combination of osimertinib and crizotinib.

We found that expression of *MET*ex14 upregulated phosphorylation of EGFR itself, presumably via cross-phosphorylation, because MET is known to interact with EGFR and drive the activity of EGFR,^20^ which resulted in blunting of the inhibition of EGFR phosphorylation by EGFR TKIs. In addition, MET inhibition restored the antagonistic effect of osimertinib on EGFR signaling. Taken together, the results suggest a complex interaction between MET and EGFR in NSCLC in the presence of EGFR TKIs and provide unique insight into potential resistance mechanisms and management strategies in *EGFR*/*MET*ex14-altered lung cancers.

In summary, *MET*ex14 is a novel mechanism of acquired resistance to EGFR TKI therapy in *EGFR*-mutant lung cancer. We show in this case that this mechanism of resistance can be effectively treated with a combination of osimertinib and crizotinib.

## Figures and Tables

**TABLE A1.**
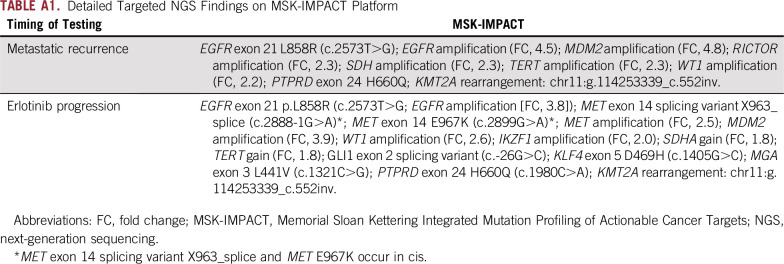
Detailed Targeted NGS Findings on MSK-IMPACT Platform
